# Development and validation of a nomogram model for predicting postoperative complications of cesarean scar pregnancy based on clinical data

**DOI:** 10.3389/fmed.2025.1652022

**Published:** 2026-01-12

**Authors:** Peiya Cai, Xiaolan Huang, Peiru Zhang

**Affiliations:** Department of Reproductive Medicine, Second Affiliated Hospital of Fujian Medical University, Quanzhou, China

**Keywords:** cesarean scar pregnancy, postoperative complications, nomogram model, risk prediction, multivariate logistic regression

## Abstract

**Background/objectives:**

Cesarean scar pregnancy (CSP) is a rare but increasingly prevalent form of ectopic pregnancy, often associated with severe postoperative complications. Current research lacks robust tools to predict these complications. This study aimed to develop and validate a clinical nomogram to assess the risk of postoperative complications in CSP patients using multidimensional clinical data.

**Methods:**

A retrospective cohort of 917 patients diagnosed with CSP between December 2015 and March 2024 was analyzed. Patients were randomly assigned to a training set (*n* = 689) and a validation set (*n* = 228). Multivariate logistic regression identified independent risk factors, which were used to construct a predictive nomogram. Model performance was evaluated by ROC curves, calibration plots, decision curve analysis, and clinical impact curves.

**Results:**

Four independent predictors of postoperative complications were identified: gestational age, interval since last cesarean section, residual myometrial thickness at the scar site, and intraoperative blood loss. The nomogram showed excellent discrimination with AUCs of 0.868 and 0.865 in the training and validation cohorts, respectively. Calibration and decision curve analyses confirmed good predictive accuracy and clinical utility.

**Conclusion:**

The developed nomogram effectively predicts postoperative complications in CSP patients and can guide early clinical interventions and personalized treatment strategies, enhancing patient safety and outcomes.

## 1 Introduction

Cesarean scar pregnancy (CSP) is a rare form of ectopic pregnancy characterized by the implantation of a fertilized ovum within the myometrial scar of a previous cesarean section (1). With the global rise in cesarean delivery rates, the incidence of CSP has increased accordingly, accounting for approximately 6% of all ectopic pregnancies (2, 3), and is closely related to the number of prior cesarean deliveries (4). CSP poses a serious threat to maternal health, as its early symptoms—typically mild vaginal bleeding or lower abdominal pain—are often nonspecific, leading to delayed diagnosis and potentially life-threatening complications such as uterine rupture or massive hemorrhage (5–10).

Although the widespread use of transvaginal ultrasonography combined with dynamic monitoring of serum *β*-human chorionic gonadotropin (β-hCG) has improved the early diagnosis of CSP (11), postoperative complications remain a major clinical concern. These include hemorrhage, infection, pelvic adhesions, and, in some cases, the need for reoperation, all of which increase hospitalization time and healthcare burden (7, 12, 13). Moreover, postoperative psychological distress—such as anxiety and depression—has increasingly been recognized as an important aspect of patient recovery (14). Previous research has largely focused on CSP pathogenesis, diagnosis, and treatment strategies (7, 15–21), including intraoperative bleeding risk and the effectiveness of different interventions (16, 22). However, few studies have systematically evaluated risk factors for postoperative complications, and existing analyses often rely on single clinical or imaging indicators, limiting individualized risk prediction (23).

Based on this, the present study aims to integrate multidimensional clinical data, including obstetric history, preoperative ultrasound imaging, surgical records, and laboratory findings, to identify independent risk factors associated with postoperative complications of CSP and develop a nomogram prediction model using multivariate logistic regression analysis. This nomogram model aimed to identify high-risk patients before surgery, guide personalized intervention strategies, lower the incidence of postoperative complications, and ultimately enhance patient outcomes.

## 2 Materials and methods

### 2.1 Study design and participants

**Figure 1:**
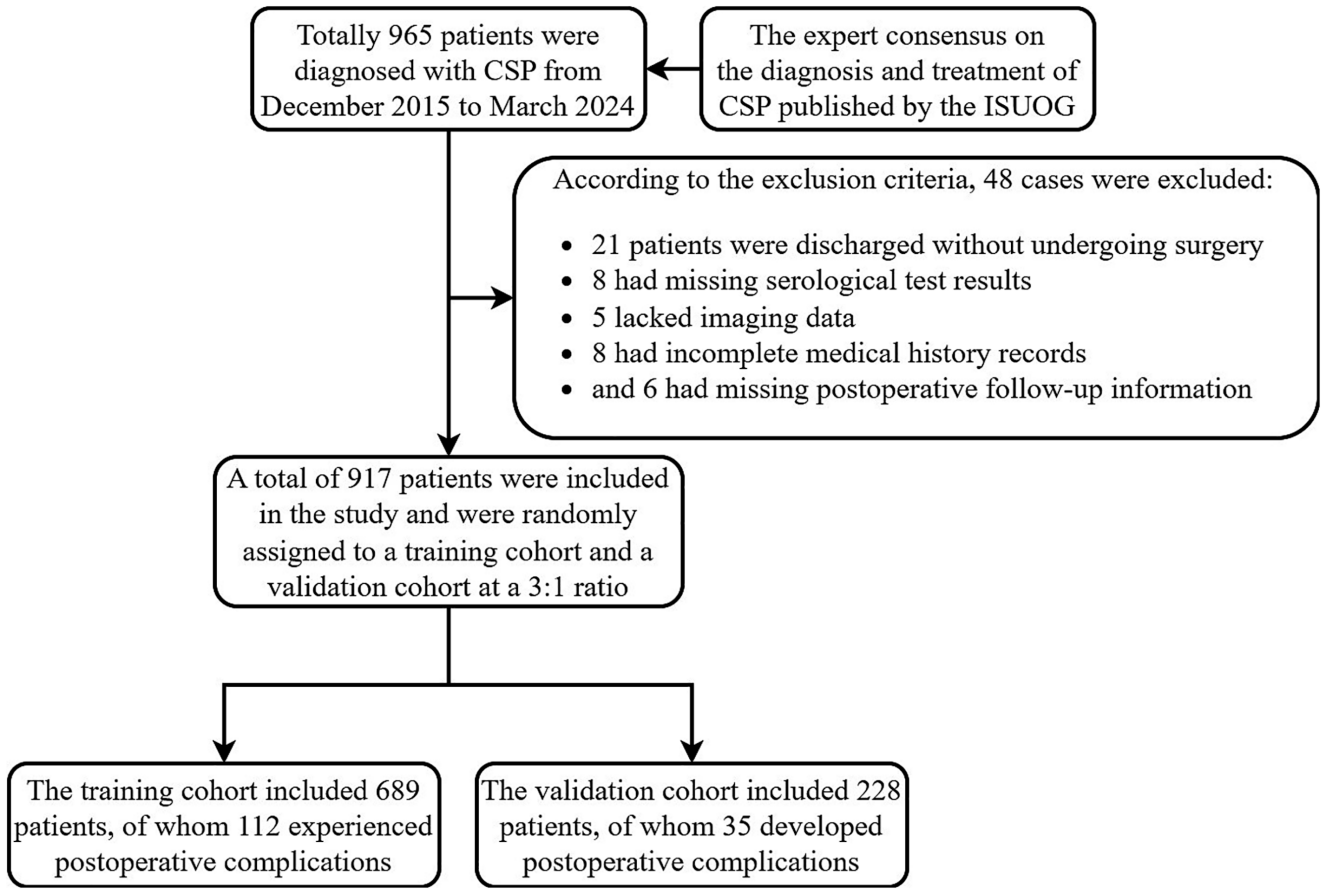
The flowchart of included and divided patients into the training group and validation group. CSP, cesarean scar pregnancy, ISUOG, International Society of Ultrasound in Obstetrics and Gynecology.

This study was conducted as a retrospective cohort analysis. A total of 965 pregnant women diagnosed with CSP at the Second Affiliated Hospital of Fujian Medical University between December 2015 and March 2024 were initially screened. Of these, 48 patients were excluded for the following reasons: 21 patients were discharged voluntarily without undergoing surgery, 8 lacked complete serological test results, 5 lacked imaging data, 8 had incomplete medical histories, and 6 were without postoperative follow-up information. After applying these criteria, 917 patients were enrolled in the study and randomly assigned to either the training cohort or the validation cohort at a 3:1 ratio using a random number table (Figure 1).

Inclusion criteria were as follows: (1) patients diagnosed with CSP according to the criteria outlined in the expert consensus on the diagnosis and treatment of CSP published by the International Society of Ultrasound in Obstetrics and Gynecology (ISUOG) (24); (2) patients who underwent surgical treatment and had complete postoperative complication records; (3) patients with complete preoperative examinations, surgical records, and postoperative follow-up data; and (4) patients aged ≥18 years.

Exclusion criteria included: (1) patients who did not undergo surgical treatment; (2) cases with an unclear history of prior uterine surgery or incomplete medical records; (3) missing key clinical data, including obstetric history, preoperative tests, surgical details, or postoperative complication follow-up; and (4) patients with severe pregnancy-related complications or known hereditary coagulation disorders.

### 2.2 Data collection and variable definitions

All data were systematically extracted and standardized from the hospital’s inpatient and outpatient electronic medical record systems, laboratory databases, and the Picture Archiving and Communication System (PACS). The variables collected included the following:

Basic demographic information: age, height, weight, body mass index (BMI), ethnicity, occupation, and education level.
Pregnancy-related history: obstetric history, history of uterine surgery, number of previous cesarean sections, Interval since the last cesarean, and pregnancy-related comorbidities.
Preoperative laboratory tests: complete blood count, coagulation parameters (prothrombin time [PT], activated partial thromboplastin time [APTT], fibrinogen, and D-dimer), liver and renal function tests, and blood glucose levels.
Preoperative imaging examinations: size and morphology of gestational sac, residual myometrial thickness at the site of cesarean scar, presence of uterine fluid, and color Doppler flow imaging (CDFI) characteristics.
Surgical information: type of surgical procedure, preoperative medical or interventional management, anesthesia method, intraoperative blood loss, requirement for intraoperative blood transfusion, and operative duration.
Postoperative complications: patients were defined as having a positive complication status if any of the following events occurred within 2 months after surgery:
Postoperative hemorrhage: bright red vaginal bleeding (indicating active bleed) or vaginal bleeding ≥ 500 mL within 24 h or clinical presentations of hypovolemia (25).
Postoperative infection: Body temperature ≥38.5 °C combined with clinical signs of pelvic infection and ultrasound or CT confirmation of pelvic abscess, consistent with general CDC principles for postoperative infection (26).
Organ injury or adhesion: uterine, bowel, or bladder perforation requiring reoperation, or presence of intestinal adhesions.
Psychological disorders: Postoperative anxiety and depression were evaluated with the validated Hospital Anxiety and Depression Scale (HADS) to reflect the biopsychosocial model of recovery in CSP patients. The anxiety (HADS-A) and depression (HADS-D) subscales were scored separately, and, consistent with prior studies (25), a score ≥11 on either subscale was used to define postoperative psychological disorders (27).

### 2.3 Statistical analysis

All collected data were organized using Microsoft Office software and analyzed with SPSS version 26.0 and R software. The Shapiro–Wilk test and histograms were used to assess the normality of continuous variables, and Levene’s test was applied to examine the homogeneity of variance. Normally distributed variables were presented as mean ± standard deviation (x̄ ± s) and compared using independent sample *t*-tests. Non-normally distributed variables were expressed as median (P25, P75) and compared using the Mann–Whitney *U* test. Categorical variables were expressed as frequencies and percentages [n (%)] and analyzed using the chi-square test or Fisher’s exact test as appropriate.

Variables with statistical significance (*p <* 0.05) in univariate logistic regression were further included in multivariate logistic regression analysis. Odds ratio (OR) and 95% confidence interval (CI) were calculated for each variable. Variables identified as independent risk factors were used to construct a nomogram model in R software based on the multivariate logistic regression coefficients. Multicollinearity among variables was assessed using the variance inflation factor (VIF) and tolerance.

The discriminative ability of the nomogram model was evaluated using the area under the receiver operating characteristic (ROC) curve (AUC). Diagnostic performance was further assessed by calculating the Youden index, sensitivity, specificity, positive predictive value, negative predictive value, positive likelihood ratio, and negative likelihood ratio. The predictive accuracy, calibration, and goodness-of-fit of the model were assessed using the Brier score, the Hosmer–Lemeshow goodness-of-fit test, and calibration curves. The Delong test was used to compare AUC values between different models to assess their generalizability across cohorts. Additionally, decision curve analysis and clinical impact curves were used to comprehensively evaluate the predictive performance, accuracy, and clinical utility of the nomogram. A two-sided *p* value < 0.05 was considered statistically significant.

## 3 Results

### 3.1 Comparison of baseline clinical characteristics between the training and validation cohorts

All eligible participants were randomly allocated into a training cohort (*n* = 689, 75%) and a validation cohort (*n* = 228, 25%). Comparative analyses were conducted for all baseline clinical variables between the two cohorts. The results showed that there were no statistically significant differences in any of the clinical characteristics (*p >* 0.05), indicating that the training and validation cohorts were comparable in terms of clinical data (Table 1).

**Table 1:** Clinical characteristics of patients in the training and validation cohorts.

Variable	Training cohort (689)	Validation cohort (228)	*t/χ*	*P* value
Age (years)	32.57 ± 4.82	32.86 ± 4.56	−0.784	0.433
Height (cm)	158.55 ± 5.15	158.18 ± 4.87	0.936	0.349
Weight (kg)	57.20 ± 9.33	57.37 ± 8.92	−0.239	0.811
BMI (kg/m^2^)	22.73 ± 3.39	22.93 ± 3.46	−0.754	0.451
Education level [*n* (%)]				
Illiterate	4 (0.6%)	2 (0.9%)	/^#^	0.7
Primary school	75 (10.9%)	20 (8.8%)		
Middle school	298 (43.3%)	102 (44.7%)		
High school	53 (7.7%)	21 (9.2%)		
Technical school	76 (11.0%)	17 (7.5%)		
Junior college	97 (14.1%)	32 (14.0%)		
Bachelor’s degree	64 (9.3%)	24 (10.5%)		
Master’s degree	22 (3.2%)	10 (4.4%)		
Gestational age (days)	51.43 ± 11.08	51.06 ± 11.58	−1.212	0.667
Gravidity, Median (P25, P75)	3 (3, 4)	3 (2.25, 4)	0.906	0.365
Miscarriages, Median (P25, P75)	1 (1, 2)	1 (1, 2)	0.717	0.474
Number of cesarean sections	2 (1, 2)	2 (1, 2)	0.000	1
Type of cesarean section [*n* (%)]				
Emergency	59 (8.6%)	26 (11.4%)	1.643	0.25
Elective	630 (91.4%)	202 (88.6%)		
Type of cesarean incision [*n* (%)]				
Transverse	679 (98.5%)	225 (98.7%)	/^#^	1
Vertical	10 (1.5%)	3 (1.3%)		
Interval since last cesarean section (years)	7 (4, 11)	7 (4, 10)	0.356	0.845
History of uterine surgery [*n* (%)]				
No	682 (99.0%)	225 (98.7%)	/^#^	0.992
Yes	7 (1.0%)	3 (1.3%)		
Gestational hypertension [*n* (%)]				
No	684 (99.3%)	224 (98.2%)	/^#^	0.328
Yes	5 (0.7%)	4 (1.8%)		
Gestational diabetes mellitus [*n* (%)]				
No	686 (99.6%)	226 (99.1%)	/^#^	0.79
Yes	3 (0.4%)	2 (0.9%)		
Endometriosis [*n* (%)]				
No	686 (99.6%)	228 (100.0%)	/^#^	0.742
Yes	3 (0.4%)	0 (0.0%)		
Maximum diameter of GS (cm)	3.09 ± 1.69	3.02 ± 1.59	0.576	0.565
Morphology of GS [*n* (%)]				
Irregular	75 (10.9%)	25 (11.0%)	0.001	1
Regular	614 (89.1%)	203 (89.0%)		
GS protrusion beyond uterine wall [*n* (%)]				
No	408 (59.2%)	137 (60.1%)	0.054	0.877
Yes	281 (40.8%)	91 (39.9%)		
Uterine fluid [*n* (%)]				
No	500 (72.6%)	173 (75.9%)	0.96	0.372
Yes	189 (27.4%)	55 (24.1%)		
Myometrial thickness^*^ (mm)	3.21 ± 1.56	3.25 ± 1.37	0.013	0.693
Vascularity of cesarean scar [*n* (%)]				
Poor	601 (87.2%)	198 (86.8%)	0.023	0.971
Rich	88 (12.8%)	30 (13.2%)		
Surgery type [*n* (%)]				
Dilation and curettage	76 (11.0%)	27 (11.8%)	/^#^	0.846
Ultrasound-guided curettage	517 (75.0%)	172 (75.4%)		
Hysteroscopic resection	50 (7.3%)	14 (6.1%)		
Laparoscopic resection	25 (3.6%)	7 (3.1%)		
Open abdominal resection	7 (1.0%)	1 (0.4%)		
Transvaginal resection	14 (2.0%)	7 (3.1%)		
Preoperative management strategy [*n* (%)]				
No specific pretreatment	138 (20.0%)	34 (14.9%)	/^#^	0.513
Intramuscular methotrexate	10 (1.5%)	2 (0.9%)		
Oral mifepristone	343 (49.8%)	122 (53.5%)		
Ultrasound-guided local methotrexate injection into GS	9 (1.3%)	2 (0.9%)		
Abdominal aortic balloon occlusion	24 (3.5%)	9 (3.9%)		
UAE	59 (8.6%)	26 (11.4%)		
UAE combined with methotrexate	106 (15.4%)	33 (14.5%)		
Anesthesia type [*n* (%)]				
Local anesthesia	412 (59.8%)	133 (58.3%)	0.152	0.755
General anesthesia	277 (40.2%)	95 (41.7%)		
Operative time (min)	32.25 ± 27.13	30.37 ± 26.10	0.916	0.36
Intraoperative blood loss (dL)	0.78 ± 1.06	0.76 ± 1.04	0.563	0.889
Intraoperative blood transfusion [*n* (%)]				
No	621 (90.1%)	204 (89.5%)	/^#^	0.815
Yes	68 (9.9%)	24 (10.5%)		
Red blood cell count (10^9^/L)	4.15 ± 1.24	4.13 ± 0.67	0.592	0.554
White blood cell count (10^9^/L)	8.14 ± 2.42	8.25 ± 2.58	−0.551	0.582
Hemoglobin level (g/L)	120.48 ± 24.94	120.78 ± 15.66	−0.169	0.866
Platelet count (10^9^/L)	241.87 ± 63.79	246.77 ± 62.38	−1.011	0.312
PT (s)	11.97 ± 1.17	12.08 ± 0.98	−1.253	0.211
APTT (s)	28.76 ± 4.62	29.18 ± 4.19	−1.206	0.228
Fibrinogen (g/L)	2.87 ± 3.00	2.88 ± 2.29	−0.033	0.973
D-dimer (μg/mL)	0.80 ± 2.75	0.51 ± 0.80	1.568	0.117
Plasma prothrombin activity (%)	96.89 ± 14.68	96.34 ± 14.95	0.496	0.62
APTT ratio	1.35 ± 5.68	1.05 ± 0.18	0.800	0.424
Thrombin time (s)	16.75 ± 6.11	16.43 ± 1.54	0.773	0.44
Alanine aminotransferase (U/L)	15.74 ± 21.30	15.39 ± 10.61	0.236	0.813
Aspartate aminotransferase (U/L)	16.51 ± 20.86	16.06 ± 9.27	0.310	0.756
Creatinine (μmol/L)	50.34 ± 42.32	48.11 ± 10.57	0.787	0.431
Blood glucose (mmol/L)	5.21 ± 4.01	5.24 ± 3.08	−0.121	0.904
Overall incidence of postoperative complications [*n* (%)]				
No	577 (83.74%)	193 (84.65%)	0.474	0.747
Yes	112 (16.26%)	35 (15.35%)		
Postoperative anxiety/depression [*n* (%)]				
No	676 (98.1%)	221 (96.9%)	0.244	0.424
Yes	13 (1.9%)	7 (3.1%)		
Postoperative hemorrhage (mL)	47.35 ± 142.40	45.53 ± 140.63	−0.651	0.451
Postoperative infection [*n* (%)]				
No	674 (97.8%)	222 (97.4%)	0.01	0.887
Yes	15 (2.2%)	6 (2.6%)		
Organ injury or adhesion [*n* (%)]				
No	648 (94.0%)	213 (93.4%)	1.555	0.854
Yes	41 (6.0%)	15 (6.6%)		
Reoperation required [*n* (%)]				
No	677 (98.3%)	222 (97.4%)	0.07	0.573
Yes	12 (1.7%)	6 (2.6%)		

BMI, body mass index; GS, gestational sac; UAE, uterine artery embolization; APTT, activated partial thromboplastin time; PT, prothrombin time; *: residual myometrial thickness at the site of cesarean scar; #: indicates values calculated using Fisher’s exact test, no corresponding test statistic is reported.

### 3.2 Analysis of independent risk factors for postoperative complications and construction of the nomogram model

#### 3.2.1 Univariate logistic regression analysis

Based on the occurrence of postoperative complications, patients in the training cohort were divided into a complication group (*n* = 112, 16.26%) and a non-complication group (*n* = 577, 83.74%). Univariate logistic regression analysis revealed 12 variables that were significantly associated with the occurrence of postoperative complications (*p <* 0.05), including gestational age, interval since last cesarean section, maximum diameter of the gestational sac, residual myometrial thickness at the site of cesarean scar, operative time, intraoperative blood loss, red blood cell count, white blood cell count, hemoglobin level, platelet count, prothrombin time, and D-dimer level (Table 2).

**Table 2:** Univariate logistic regression analysis based on training group.

Variable	OR (95%CI)	*P* value
Age	0.991 (0.950–1.033)	0.670
Height	1.001 (0.962–1.041)	0.958
Weight	1.010 (0.989–1.031)	0.370
BMI	1.024 (0.966–1.086)	0.417
Education level	1.656 (0.748–3.666)	0.214
Gestational age	1.086 (1.065–1.107)	**<0.001**
Gravidity	0.988 (0.860–1.134)	0.860
Miscarriages	0.978 (0.831–1.149)	0.785
Number of cesarean sections	0.929 (0.654–1.320)	0.682
Type of cesarean section	0.047 (0.364–7.930)	0.880
Type of cesarean incision	0.895 (0.865–1.427)	0.976
Interval since last cesarean section	0.835 (0.787–0.885)	**<0.001**
History of uterine surgery	0.857 (0.102–7.186)	0.887
Gestational hypertension	1.291 (0.143–11.649)	0.820
Gestational diabetes mellitus	2.590 (0.233–28.782)	0.439
Endometriosis	0.052 (0.362–8.524)	0.980
Maximum diameter of GS	1.446 (1.289–1.622)	**<0.001**
Morphology of GS	1.478 (0.714–3.061)	0.293
GS protrusion beyond uterine wall	1.014 (0.672–1.532)	0.946
Uterine fluid	1.249 (0.804–1.939)	0.323
Myometrial thickness^*^	0.510 (0.417–0.624)	**<0.001**
Vascularity of cesarean scar	0.791 (0.415–1.509)	0.477
Surgery type	1.271 (0.464–3.479)	0.641
Preoperative management strategy	1.429 (0.827–1.870)	0.296
Anesthesia type	1.243 (0.827–1.870)	0.296
Operative time	1.018 (1.012–1.025)	**<0.001**
Intraoperative blood loss	2.491 (2.009–3.088)	**<0.001**
Intraoperative blood transfusion	1.125 (1.005–1.652)	0.362
Red blood cell count	0.453 (0.318–0.646)	**<0.001**
White blood cell count	1.097 (1.013–1.189)	**0.023**
Hemoglobin level	0.977 (0.966–0.988)	**<0.001**
Platelet count	0.996 (0.993–1.000)	**0.032**
PT	1.200 (1.012–1.423)	**0.036**
APTT	1.005 (0.961–1.050)	0.836
Fibrinogen	1.035 (0.981–1.092)	0.204
D-dimer	1.098 (1.027–1.174)	**0.006**
Plasma prothrombin activity	0.992 (0.980–1.005)	0.248
APTT ratio	1.016 (0.990–1.043)	0.236
Thrombin time	0.925 (0.840–1.020)	0.119
Alanine aminotransferase	0.997 (0.985–1.010)	0.673
Aspartate aminotransferase	0.996 (0.981–1.012)	0.626
Creatinine	0.979 (0.957–1.001)	0.060
Blood glucose	0.984 (0.918–1.055)	0.653

BMI, body mass index; GS, gestational sac; APTT, activated partial thromboplastin time; PT, prothrombin time; OR, odds ratio; 95%CI, 95% confidence interval; *: residual myometrial thickness at the site of cesarean scar. Bold values indicate statistical significance (*P* < 0.05).

#### 3.2.2 Multivariate logistic regression analysis

Variables that were statistically significant in the univariate analysis were included in the multivariate logistic regression analysis. The results showed that gestational age, interval since the last cesarean section, residual myometrial thickness at the site of cesarean scar, and intraoperative blood loss were identified as independent risk factors for postoperative complications in CSP patients (*p <* 0.05) (Table 3).

**Table 3:** Multivariate logistic regression analysis based on the data of training group.

Variable	*β* value	Wald z value	OR (95% CI)	*P* value
Gestational age (days)	0.062	4.636	1.064 (1.037–1.093)	**<0.001**
Interval since last cesarean (years)	−0.264	−6.332	0.768 (0.705–0.830)	**<0.001**
Myometrial thickness (mm)^*^	−0.463	−3.813	0.629 (0.429–0.792)	**<0.001**
Intraoperative blood loss (dL)	0.718	4.693	2.050 (1.544–2.818)	**<0.001**

OR, odds ratio; 95%CI, 95% confidence interval; *: residual myometrial thickness at the site of cesarean scar. Bold values indicate statistical significance (*P* < 0.05).

#### 3.2.3 Multicollinearity assessment

Multicollinearity was assessed among the four independent risk factors for postoperative complications in CSP. The results showed that all variables had variance inflation factors (VIFs) less than 10 and tolerances greater than 0.1, indicating the absence of significant multicollinearity (Table 4).

**Table 4:** Multicollinearity test of independent risk factors for postoperative complications of cesarean scar pregnancy.

Variable	VIF	Tolerance
Gestational age (days)	1.231	0.812
Interval since last cesarean (years)	1.214	0.824
Myometrial thickness (mm)^*^	1.087	0.920
Intraoperative blood loss (mL)	1.512	0.661

VIF, variance inflation factors; *: Residual myometrial thickness at the site of cesarean scar.

### 3.3 Construction of the nomogram model

**Figure 2:**
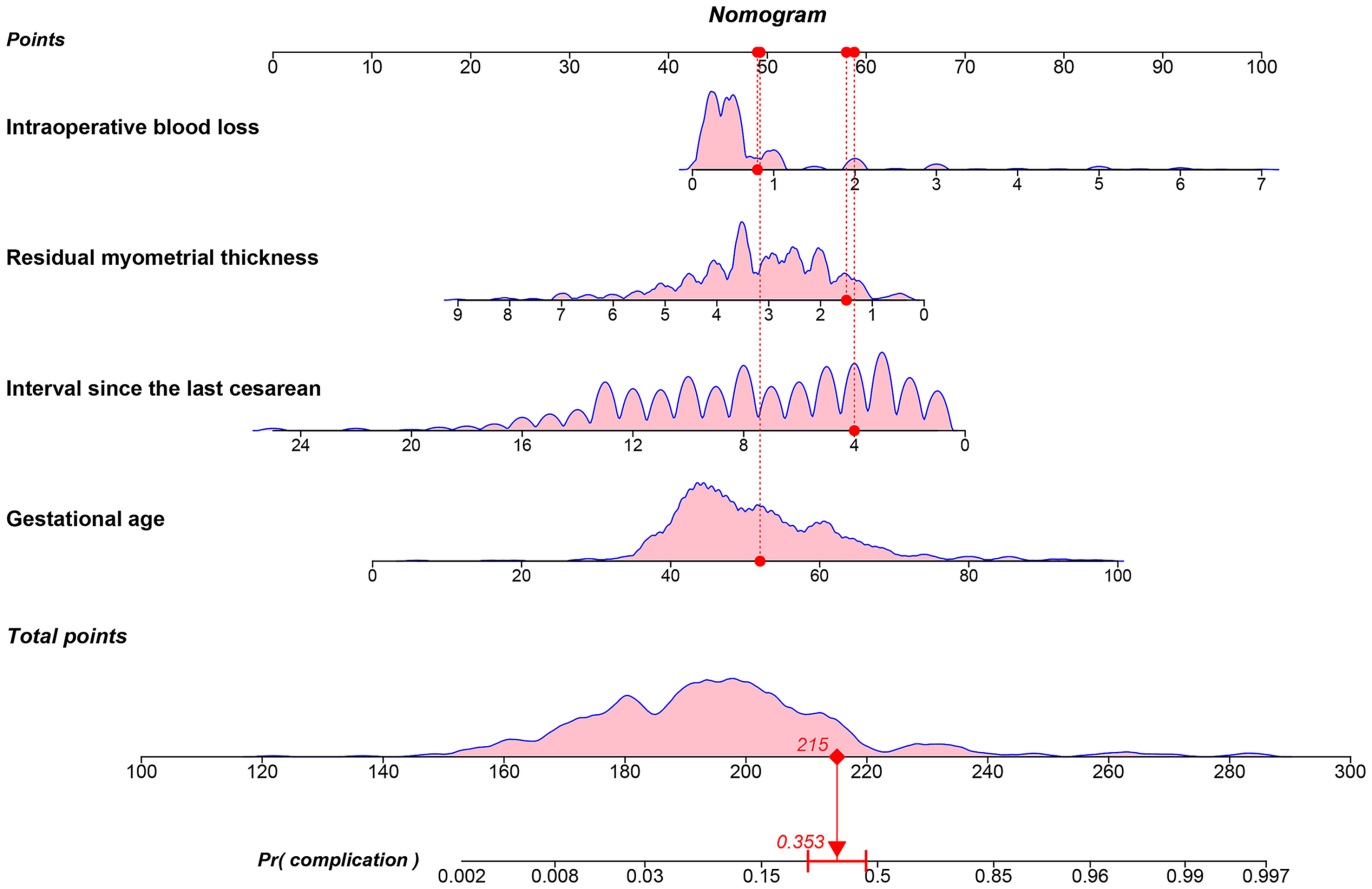
Nomogram model for predicting postoperative complications of cesarean scar pregnancy based on clinical data. The red arrow indicates a CSP patient with 4 years since the last cesarean section, a myometrial thickness of 1.5 mm, a gestational age of 52 days, and an intraoperative blood loss of 0.8 dL. The total score is 215 points, and based on the nomogram model, the probability of developing postoperative complications is 0.353. Residual myometrial thickness: Residual myometrial thickness at the site of cesarean scar.

Based on the results of multivariate logistic regression, four independent risk factors for postoperative complications (gestational age, interval since the last cesarean section, residual myometrial thickness at the site of cesarean scar, and intraoperative blood loss) were incorporated into R software using the *rms* package to construct a nomogram model for predicting postoperative complications in CSP patients. The resulting predictive model is illustrated in Figure 2. The chi-square statistic of the logistic regression model was 101.44 (*p <* 0.001), indicating a statistically significant relationship between the independent variables and the risk of postoperative complications.

The risk prediction equation derived from the logistic regression model is as follows:

Risk of postoperative complications in CSP = −0.47341 × myometrial thickness between gestational sac and bladder (mm) + 0.0681 × gestational age (days) − 0.25308 × interval since last cesarean section (years) + 0.74332 × intraoperative blood loss (dL) − 3.01909.

### 3.4 Evaluation of model performance

#### 3.4.1 Accuracy and discrimination

**Figure 3:**
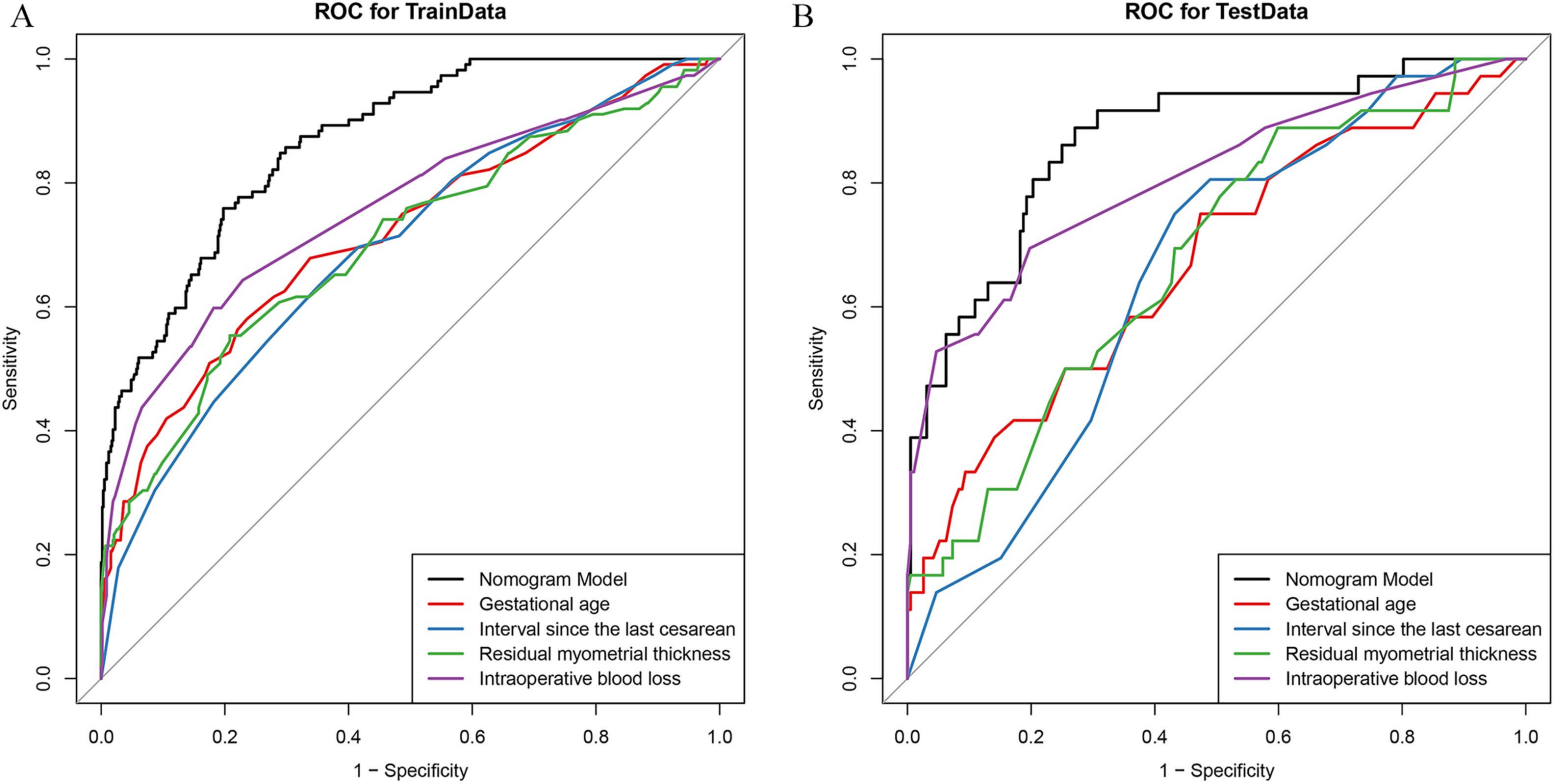
ROC curves of the nomogram model and the 4 independent risk factors in predicting postoperative complications of cesarean scar pregnancy patients in training group **(A)** and validation group **(B)**. The AUC of the nomogram model for predicting postoperative complications in CSP patients was 0.868 (95% CI = 0.833–0.901) in the training cohort **(A)** and 0.865 (95% CI, 0.801–0.934) in the validation cohort **(B)**. Both were significantly higher than the AUC of the 4 independent risk factors used alone. *: Residual myometrial thickness at the site of cesarean scar.

The occurrence of postoperative complications was defined as the outcome variable (assigned as 1 for “yes” and 0 for “no”). Based on the previously established logistic regression equation, nomogram prediction models were constructed for both the training and validation cohorts. Receiver operating characteristic (ROC) curve analysis was conducted to evaluate model performance. The area under the ROC curve (AUC) for the nomogram model was 0.868 (95% CI = 0.833–0.901) in the training cohort and 0.865 (95% CI, 0.801–0.934) in the validation cohort. DeLong test indicated that the AUCs of the nomogram models in both cohorts were significantly higher than those of the individual predictors (*p <* 0.01) (Figure 3).

Furthermore, DeLong test revealed no statistically significant difference in AUC between the training and validation cohorts (D = −0.020, *p* = 0.984), indicating that the model had stable discriminative ability across datasets. In addition, the nomogram demonstrated favorable predictive accuracy in both cohorts, with the Youden index, sensitivity, and specificity being 0.561, 0.759, and 0.802 in the training cohort, and 0.618, 0.889, and 0.719 in the validation cohort, respectively (Table 5).

**Table 5:** The accuracy of predicting postoperative complications in cesarean scar pregnancy patients using a nomogram model.

Indicator	Cohort	
	Training	Validation
AUC (95% CI)	0.868 (0.833–0.901)	0.865 (0.801–0.934)
Youden Index	0.561	0.618
Sensitivity	0.759	0.889
Specificity	0.802	0.719
Positive predictive value	0.793	0.766
Negative predictive value	0.769	0.868
Positive likelihood ratio	3.841	3.282
Negative likelihood ratio	0.300	0.152

AUC, area under the curve; 95%CI, 95% confidence interval.

#### 3.4.2 Calibration

**Figure 4:**
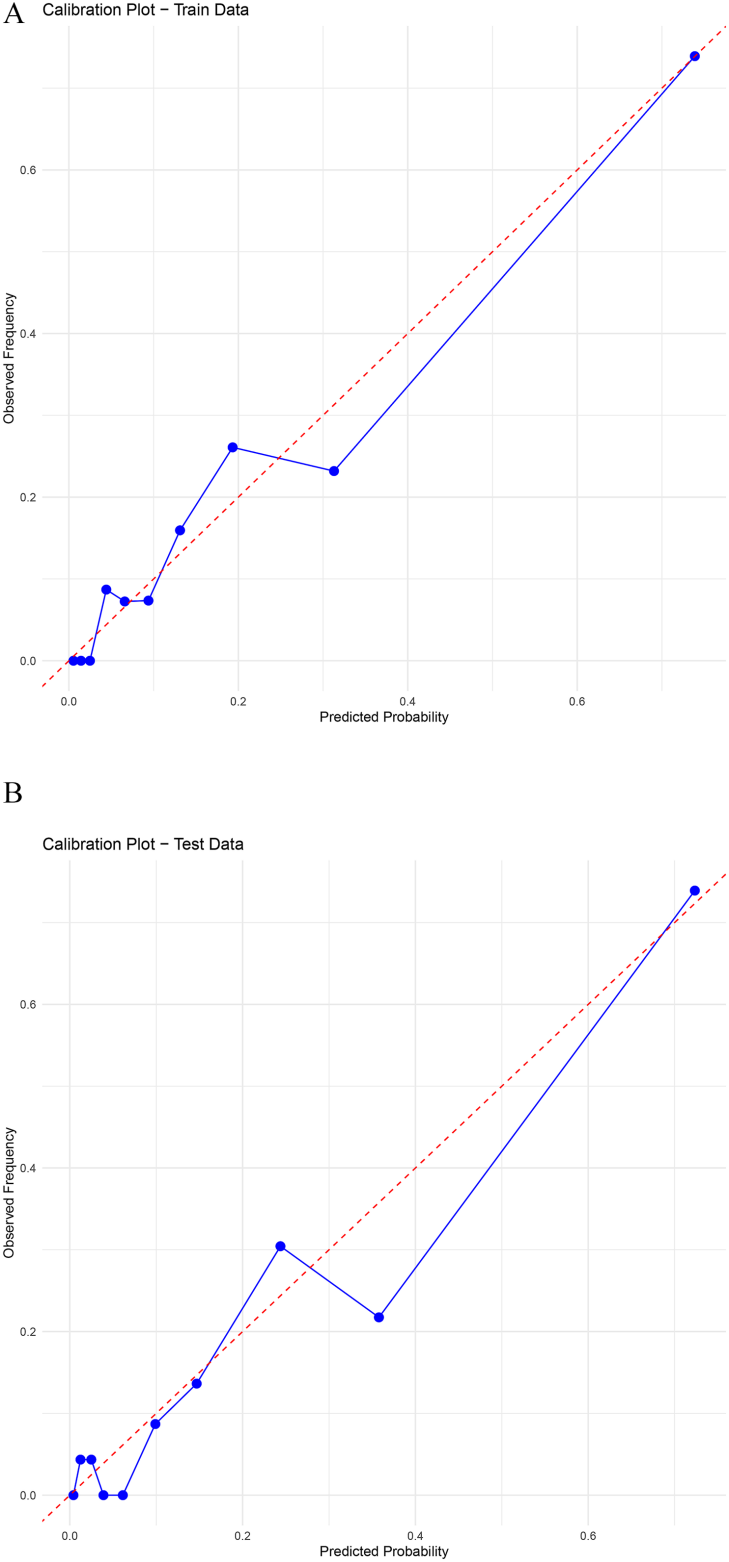
Calibration curves of the nomogram model for the training group **(A)** and validation group **(B)**. The blue solid line represents the calibration curve of the model, while the red dashed line represents the ideal calibration curve. The calibration curves for the training group **(A)** and validation group **(B)** were both close to the ideal calibration curve.

The nomogram model demonstrated good predictive performance, with Brier scores of 0.089 in the training cohort and 0.086 in the validation cohort. In addition, the Hosmer–Lemeshow goodness-of-fit test yielded *p*-values of 0.195 and 0.520 for the training and validation cohorts, respectively, both greater than 0.05, suggesting a good model fit without evidence of overfitting or underfitting. Calibration curves further demonstrated that the predicted probabilities from the nomogram closely matched the actual observed outcomes in both cohorts (Figure 4), confirming the model’s high calibration performance.

##### 3.4.2.1 Decision curve analysis

**Figure 5:**
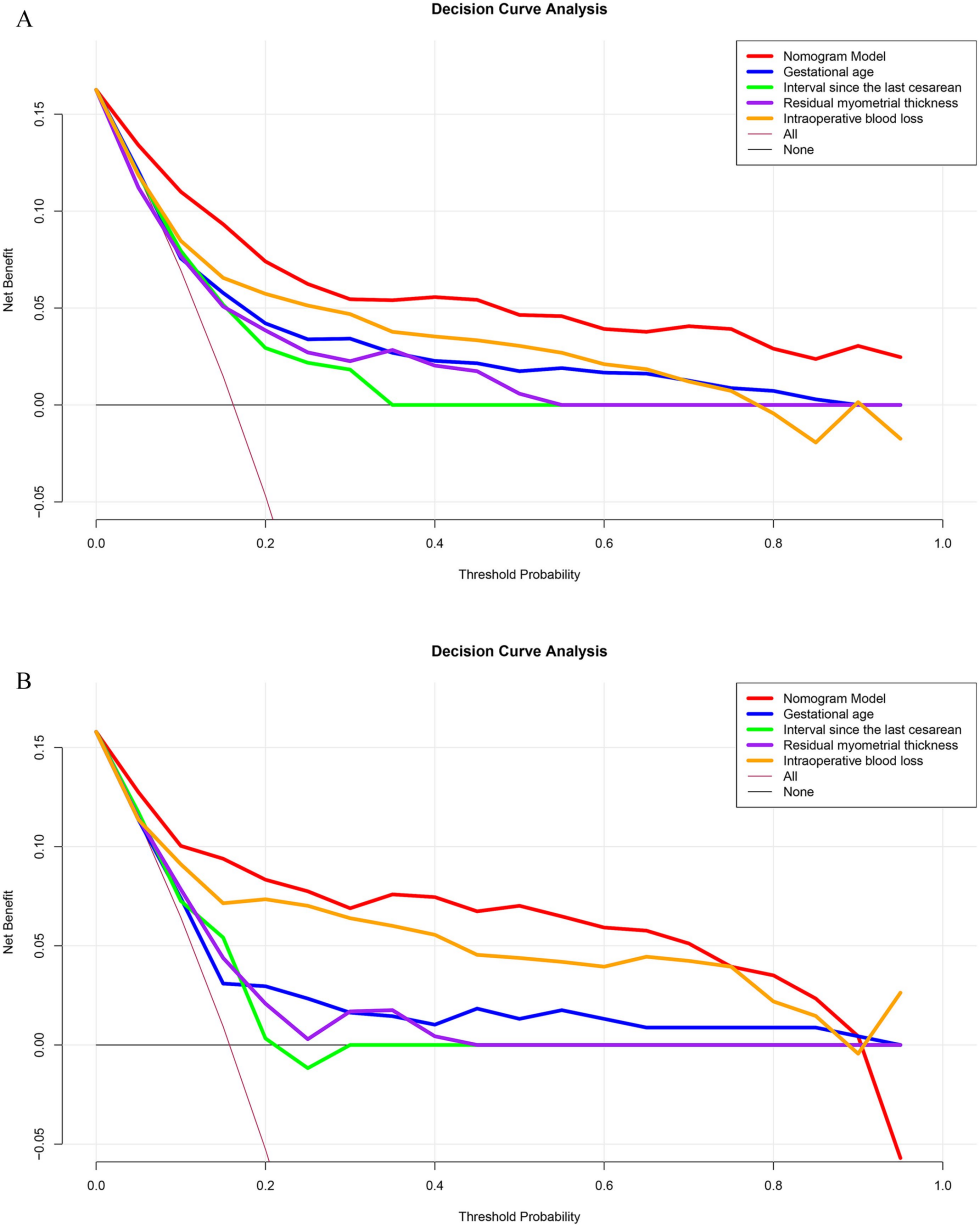
Decision curve analysis curves for the nomogram model and the 4 independent risk factors in predicting postoperative complications of cesarean scar pregnancy patients in training group **(A)** and validation group **(B)**. In the training group **(A)** and validation group **(B)**, the nomogram model outperformed the use of the 4 independent risk factors alone in terms of threshold probability range and net benefit (*p <* 0.05 for all). *: Residual myometrial thickness at the site of cesarean scar.

To further evaluate the clinical applicability of the nomogram at various intervention threshold probabilities, decision curve analysis was performed for both the training and validation cohorts (Figure 5). In the training cohort, the model demonstrated a clear net benefit across a wide range of threshold probability (Figure 5A), outperforming the “All” and “None” strategies. Similarly, the model showed favorable decision performance in the validation cohort (Figure 5B), indicating strong potential for clinical implementation. Moreover, compared with models based on single predictors, the nomogram consistently achieved higher net benefits in both cohorts, reinforcing the predictive superiority and clinical value of the integrated multivariable model. In this study, the “All” curve represents the hypothetical scenario in which all CSP patients are assumed to experience postoperative complications and receive intervention, while the “None” curve assumes that no patient experiences complications and no intervention is administered.

##### 3.4.2.2 Clinical impact curve analysis

**Figure 6:**
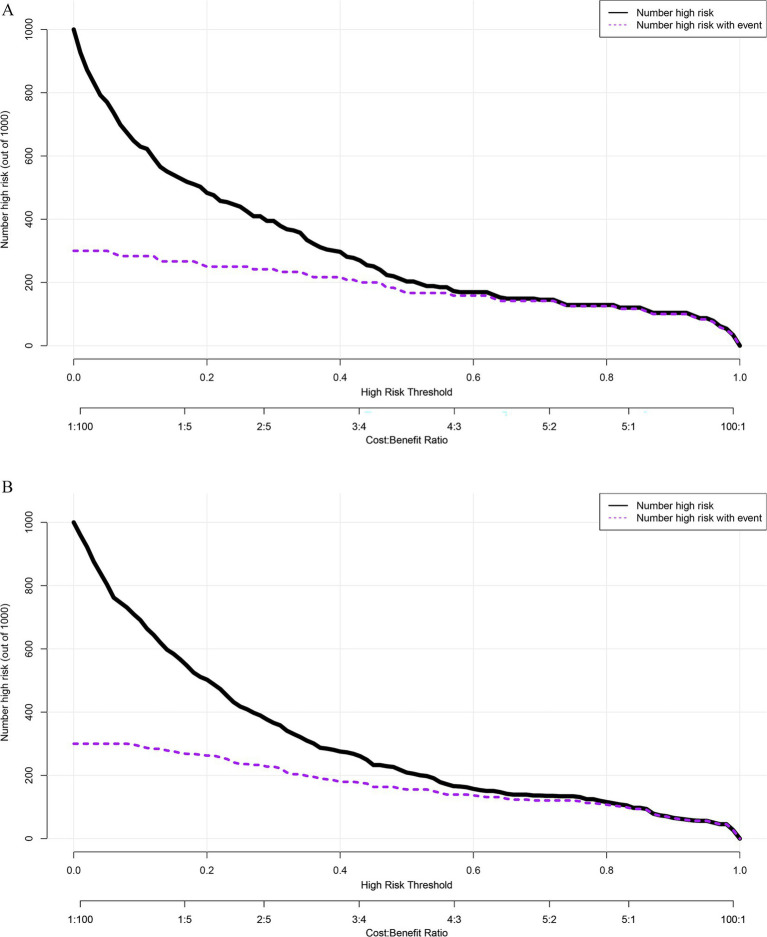
Clinical impact curve of the nomogram model for predicting postoperative complications in cesarean scar pregnancy patients in the training group **(A)** and validation group **(B)**. The black solid line represents the number of individuals identified as high-risk by the model at each domain probability threshold. The purple dashed line represents the actual number of high-risk patients at each threshold probability. The nomogram model shows that in the training group **(A)** and validation group, when the threshold probability is greater than 60%, for the predicted score, the “number of high-risk patients marked by the model” line closely aligns with the “actual number of high-risk patients” line. This indicates that the nomogram model can accurately identify high-risk CSP patients for postoperative complications, confirming its high clinical net benefit rate.

Clinical impact curve analysis was employed to assess the ability of the nomogram model to identify CSP patients at high risk of postoperative complications (Figure 6). The results demonstrated that in both the training cohort (Figure 6A) and the validation cohort (Figure 6B), when the threshold probability exceeded 60%, the number of patients predicted by the model as high risk closely aligned with the number of patients who actually experienced complications. This indicates that the nomogram possesses strong clinical discriminative ability and can accurately identify true high-risk individuals in the higher-risk probability range, thereby offering substantial clinical net benefit.

## 4 Discussion

In this study, a nomogram prediction model for postoperative complications of CSP was successfully developed based on a retrospective cohort analysis utilizing multidimensional clinical data. The model incorporated four independent risk factors—gestational age, interval since the last cesarean section, residual myometrial thickness at the site of cesarean scar, and intraoperative blood loss—and demonstrated effective predictive ability for assessing the risk of postoperative complications in CSP patients. Psychological outcomes such as anxiety and depression were also included, reflecting the growing recognition of mental health as an essential component of postoperative recovery in CSP patients. Furthermore, the nomogram exhibited strong discriminatory power and good calibration performance, and showed high predictive accuracy and practical utility in clinical settings.

The nomogram model indicates that a longer gestational age was associated with an increased risk of postoperative complications in CSP patients. Previous studies have primarily focused on the relationship between gestational age and intraoperative blood loss, without investigating its association with postoperative complications (28, 29). The elevated risk observed with increasing gestational duration may be attributed to several physiological and anatomical factors. As the embryo grows, its increasing requirement for blood flow may exceed the limited vascular capacity of the scar tissue, thereby predisposing the area to ischemia or hemorrhage due to inadequate perfusion (30, 31). In addition, the uterine expansion during pregnancy may place mechanical stress on the cesarean scar, where the tissue is less elastic and structurally rigid, making it less capable of adapting to uterine enlargement, thereby increasing the risk of scar dehiscence or even uterine rupture (32). Moreover, rising levels of *β*-hCG and progesterone may enhance the invasive potential of trophoblastic tissue into the scar site, further increasing the risk of complications. Collectively, these physiological and structural changes associated with prolonged gestation may explain the observed increase in complication rates with advancing gestational age in CSP.

Moreover, a shortened interval since the last cesarean section was linked to a higher risk of postoperative complications in CSP. Inadequate healing of the uterine scar due to insufficient time between pregnancies may compromise scar strength, increasing the likelihood of structural failure and associated complications (30, 33). Under such circumstances, embryo implantation at an incompletely healed scar may impair development and elevate the risk of hemorrhage or uterine rupture due to poor elasticity and limited blood supply (30). In addition, insufficient time between surgeries may hinder complete revascularization at the scar site, resulting in suboptimal blood and oxygen supply to the embryo, thereby increasing the risk of abnormal development and complications (31, 33). Taken together, early conception after cesarean delivery elevates the risk of CSP-related complications, primarily due to inadequate scar healing, poor perfusion, and incomplete recovery of the uterine myometrium. Our findings further support the strong link between interpregnancy interval and postoperative outcomes, highlighting the need for evidence-based recommendations on optimal spacing to minimize adverse events.

This study also found that a thinner residual myometrial thickness at the site of cesarean scar was significantly associated with a higher risk of postoperative complications in CSP patients. Similar findings have been reported by other researchers, who observed that a thinner lower anterior uterine wall increases the likelihood of severe hemorrhage (28, 29). A structurally weakened myometrium, with reduced strength and elasticity, may fail to support normal embryonic development, increasing the likelihood of scar rupture and hemorrhage (30, 32). Moreover, reduced myometrial thickness is often accompanied by impaired blood supply, which limits the delivery of oxygen and nutrients to the gestational sac. This may lead to local ischemia and increase the risk of adverse outcomes such as ectopic pregnancy or miscarriage (31, 33). In addition, thin myometrium may facilitate abnormal implantation with excessive trophoblastic invasion into scar tissue, elevating the risk of morbidly adherent placenta, including placenta accreta (34, 35). Thus, impaired myometrial integrity is a key factor in pregnancy progression and significantly contributes to the elevated risk of postoperative complications in CSP cases.

Finally, greater intraoperative blood loss was found to be associated with an increased risk of postoperative complications in CSP patients. While most existing studies have focused on identifying the risk factors for intraoperative hemorrhage (29, 36), few have specifically investigated the relationship between intraoperative blood loss and postoperative complications. We hypothesize that excessive intraoperative blood loss may indicate more extensive uterine injury. Severe vascular disruption or laceration at the cesarean incision site can compromise scar integrity, leading to incomplete healing and reduced myometrial strength and elasticity and increasing the risk of postoperative complications (32). In addition, massive hemorrhage can result in anemia and hypotension, which may compromise uterine perfusion and scar repair (37). Furthermore, substantial blood loss often prolongs the duration and complexity of surgery, increasing the likelihood of psychological consequences such as postoperative anxiety or depression (38). Taken together, excessive intraoperative bleeding may impair uterine healing, reduce tissue perfusion, and increase psychological burden, collectively heightening the risk of postoperative complications in CSP patients.

The nomogram model developed in this study demonstrated significantly better discriminatory ability of the integrated multivariable model in predicting postoperative complications compared to each individual risk factor. An AUC above 0.80 is generally considered indicative of good discriminative performance, suggesting that the model can reliably distinguish between high-risk and low-risk patients. Moreover, DeLong test showed no significant difference in AUC between the two cohorts, indicating consistent performance across datasets and confirming the model’s robustness and generalizability. The model also exhibited favorable diagnostic accuracy, goodness-of-fit, and calibration in both cohorts.

Decision curve analysis further demonstrated that the nomogram yielded a consistent net benefit across a broad range of threshold probabilities, with particularly strong performance in the intermediate-risk range where clinical decision-making is most challenging. This suggests that the model offers not only statistically significant predictive value but also practical utility in clinical decision-making. Compared with single-variable models, the integrated nomogram more accurately stratifies patient risk, thereby supporting personalized postoperative care and more efficient allocation of healthcare resources. Clinical impact curve analysis further validated the model’s utility across various threshold levels, especially its precision in identifying high-risk patients. When a higher clinical intervention threshold (≥60%) was applied, the nomogram not only effectively identified patients at high risk of complications but also minimized false-positive rates. This enhances its applicability in real-world clinical settings by facilitating targeted postoperative interventions and improving the efficiency of risk-based management. This nomogram could be further implemented as a web-based calculator or mobile application, allowing clinicians to input patient-specific variables and instantly estimate the risk of postoperative complications in CSP. Such digital integration would enable real-time risk stratification and personalized perioperative management, thereby enhancing clinical decision-making and optimizing patient outcomes.

In routine practice, this nomogram can serve as a simple bedside tool to estimate an individual patient’s risk of postoperative complications using routinely available clinical variables. The predicted risk can help guide triage and allocation of perioperative resources, as well as support more personalized preoperative counseling and expectation management. In addition, once integrated into an electronic calculator or clinical pathway, the model may assist postoperative surveillance by flagging women at higher risk who warrant closer follow-up. The nomogram is intended to complement, rather than replace, clinical judgment.

This study has several limitations. First, it was conducted in a single tertiary center in China, which may limit the generalizability of the model to other settings. Second, the retrospective design precludes firm causal inferences. Third, only women who underwent surgical management for CSP were included, so medically or expectantly managed cases were not captured, which may introduce selection bias. Finally, postoperative psychological outcomes were assessed using HADS alone and may be influenced by unmeasured factors such as pre-existing mental disorders, social support, and socioeconomic status.

## 5 Conclusion

This study successfully developed a nomogram model for predicting postoperative complications in CSP based on gestational age, interval since the last cesarean section, residual myometrial thickness at the site of cesarean scar, and intraoperative blood loss. The model demonstrated good predictive accuracy and clinical utility, offering a valuable tool for early identification of high-risk patients. It may assist in optimizing surgical planning and reducing postoperative complications, thereby showing great potential for clinical application and widespread implementation.

## Data Availability

The raw data supporting the conclusions of this article will be made available by the authors, without undue reservation.
